# Alternative splicing remodeling and splicing factor regulatory networks define immune-associated molecular heterogeneity in dilated cardiomyopathy

**DOI:** 10.3389/fbinf.2026.1934275

**Published:** 2026-09-04

**Authors:** Jia Li, Yiyu He, Xianxian Zhao, Zhifu Guo, Jinchao Song, Xiaowei Song

**Affiliations:** 1 Department of Cardiovascular Medicine, Changhai Hospital, Naval Medical University, Shanghai, China; 2 Department of Anesthesiology, Shidong Hospital of Shanghai, University of Shanghai for Science and Technology, Shanghai, China

**Keywords:** alternative splicing, dilated cardiomyopathy, machine learning, molecular heterogeneity, splicing factor

## Abstract

Dilated cardiomyopathy (DCM), a progressive cardiac disorder marked by ventricular dilatation and systolic dysfunction, involves a complex interplay between genetic and immune dysregulation. However, how alternative splicing remodeling contributes to its molecular heterogeneity remains poorly understood. Here, we performed an integrated transcriptomic analysis to characterize DCM-associated alternative splicing landscapes and their regulation by splicing factor networks. We identified widespread differential alternative splicing (DAS) events in DCM, with affected genes significantly enriched in cardiac contraction, sarcomere organization, calcium handling, extracellular matrix remodeling, and protein metabolism. Representative splicing events in key cardiac genes, including *TTN*, *RYR2*, *LDB3*, and *FLNA*, exhibited distinct junction usage and percent spliced in (PSI) alterations between DCM and control samples, suggesting functionally relevant transcript isoform remodeling. By integrating splicing factor expression profiles with DAS event PSI values, we constructed a characteristic SF-AS regulatory network and identified five key splicing factors—*MBNL1*, *NOVA1*, *RBM25*, *RBM5*, and *SF1*—as candidate tissue-level biomarkers of DCM-associated splicing remodeling. Molecular subtype analysis further revealed distinct DCM subgroups characterized by divergent immune infiltration patterns, pathway activities, and hub gene signatures. Machine learning-based identification and external validation supported the diagnostic potential of this five-splicing-factor signature. Collectively, this study deciphers coordinated splicing factor-associated alternative splicing remodeling in DCM, providing deeper insights into the immune-associated molecular heterogeneity and potential regulatory mechanisms underlying disease progression.

## Introduction

Dilated cardiomyopathy (DCM) is a prevalent heart muscle disease characterized by ventricular dilation and systolic dysfunction, often leading to heart failure and an increased risk of sudden cardiac death, leading to significant morbidity and mortality worldwide (Schultheiss et al., 2019). DCM represents a significant proportion of pediatric cardiomyopathies, accounting for approximately 60%, with the highest incidence observed within the first year of life (Towbin et al., 2006). While the etiology of DCM has been identified in some cases, many remain idiopathic. The causes of DCM are multifactorial, encompassing both acquired and genetic factors. Acquired etiologies include infectious agents, medications, toxins, alcohol, nutritional deficiencies, peripartum conditions, as well as autoimmune, metabolic, and endocrine disorders (Maron et al., 2006; Pinto et al., 2016). Currently, while the genetic complexity underlying DCM remains incompletely elucidated, known genetic causes encompass mutations in over 60 distinct genes (Fatkin et al., 2017). These DCM-related genes encode a diverse array of proteins, such as cytoskeletal components, sarcomeric proteins, Ca^2+^ regulatory molecules, ion-channel subunits, and desmosomal proteins (Pinto et al., 2016).

Cardiac ischemia triggers cardiomyocyte apoptosis and necrosis, which activate tissue-resident immune cells. These activated cells release proinflammatory cytokines, recruit circulating leukocytes to the ischemic myocardium, and initiate localized inflammation (Frangogiannis and Entman, 2005). Concurrently, chemokine secretion by immune cells promotes macrophage and neutrophil infiltration, enabling phagocytic clearance of cellular debris (Frangogiannis, 2006). Although immunocyte infiltration is essential for ischemic injury resolution (Matsumoto et al., 2011), its contribution to myocardial inflammation in nonischemic DCM remains uncharacterized. These remodeling processes provide a broader disease context in which transcriptomic and alternative-splicing dysregulation may contribute to DCM progression.

Emerging clinical and experimental evidence underscores the pivotal role of immune dysregulation in shaping the clinical trajectory of DCM (Felix et al., 2015; Zhao and Fu, 2018). Pioneering epidemiological investigations, such as the longitudinal cohort study by Lipshultz et al. (1998), demonstrated a direct association between immune dysfunction and DCM pathogenesis. Contemporary analyses further highlight sex-specific immunological risks: female patients with concurrent autoimmune disorders exhibit elevated cardiovascular morbidity and heart transplantation rates compared to their non-autoimmune counterparts (Verdonschot et al., 2021). Conversely, therapeutic interventions targeting immune modulation, such as immunoadsorption therapy (Yoshikawa et al., 2016) and intravenous immune globulin administration (McNamara et al., 2001), have demonstrated benefits in alleviating symptoms and enhancing exercise tolerance in DCM patients.

Alternative splicing serves as a critical post-transcriptional mechanism for proteomic diversification, with splicing factors acting as fundamental regulators of this process (Lee and Rio, 2015; Koch, 2017). Notably, dysregulation of these RNA-binding proteins has been clinically associated with various pathological states, particularly cardiomyopathies where aberrant splicing patterns directly contribute to myocardial dysfunction (Radke et al., 2021). Emerging evidence further implicates immune dysregulation in dilated cardiomyopathy (DCM) pathogenesis, with recent transcriptomic studies revealing significant interactions between alternative splicing networks and inflammatory signaling pathways (Saito et al., 2018). This mechanistic convergence suggests dual therapeutic potential through targeted modulation of both spliceosomal activity and immune responses in DCM management.

Recent studies have highlighted the role of splicing factors in the regulation of gene expression and the development of heart diseases. For instance, mutations in splicing factors have been associated with familial DCM, suggesting a mechanistic link between AS dysregulation and cardiac dysfunction (Yamada and Nomura, 2021; McNally and Mestroni, 2017). The RNA-binding motif protein 20 (RBM20), a cardiac splicing regulator, was first implicated in DCM through pathogenic mutations identified in 2009 (Nishiyama et al., 2022). Moreover, the immune system serves as a pivotal role in the progression of DCM, with immune-mediated inflammation contributing to myocardial damage and disease severity (Nussinovitch and Shoenfeld, 2013).

Despite advancements in our understanding of DCM, the interplay between splicing factor-mediated regulatory networks and immunological characteristics in DCM has not been comprehensively explored. In this study, we aim to reveal the splicing factor-mediated regulatory networks and immunological characteristics in DCM through integrative bioinformatics and machine learning. This comprehensive investigation not only holds the promise of providing novel insights into the molecular pathogenesis of DCM but also has the potential to pave the way for the development of more effective diagnostic feature and therapeutic strategies, ultimately improving the clinical management of patients with this debilitating heart disease.

## Materials and methods

### Data collection and processing

**FIGURE 1:**
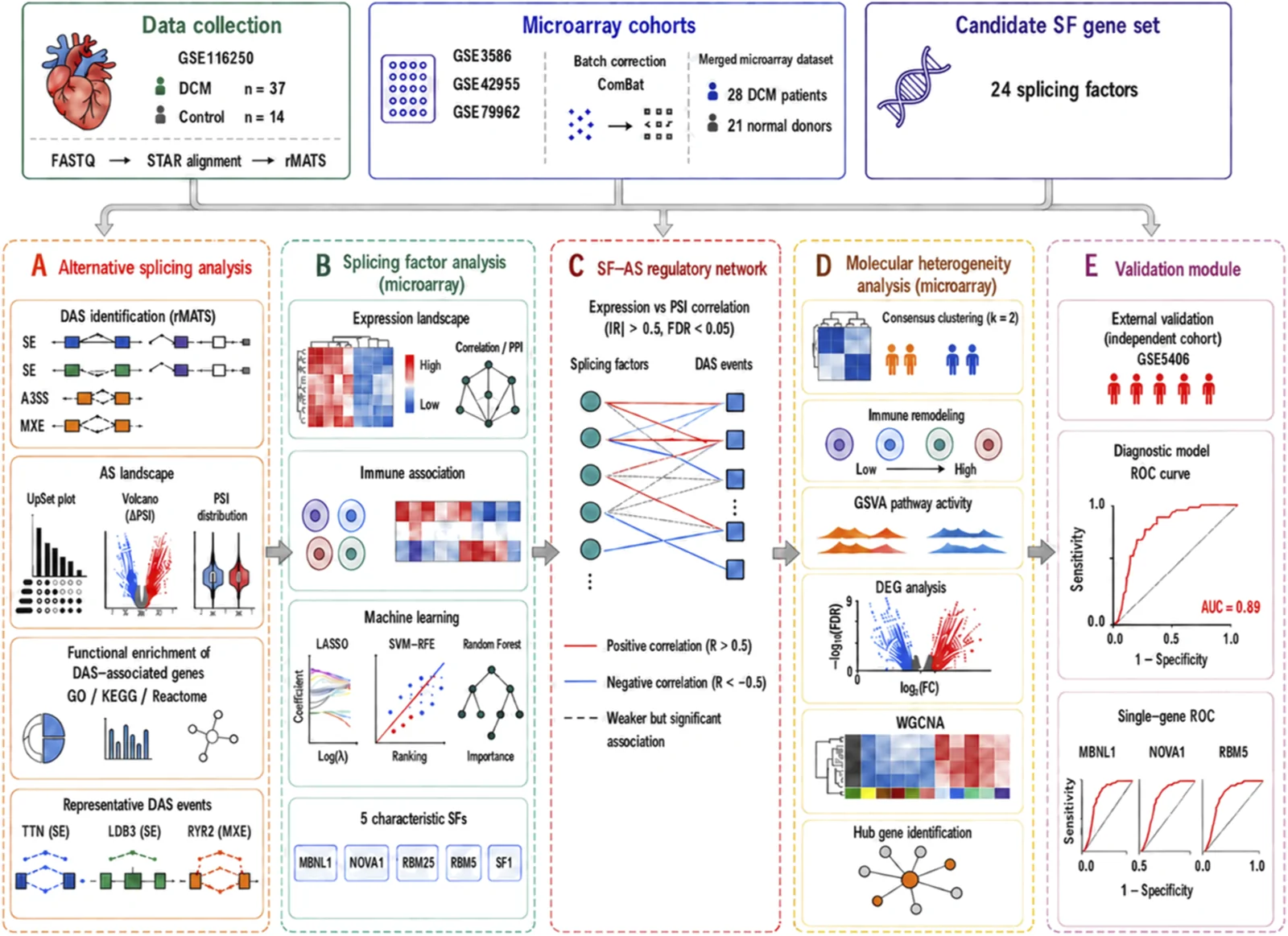
Schematic overview of the integrated analytical workflow. DCM, dilated cardiomyopathy; DAS, differential alternative splicing; SF, splicing factor; PSI, percent spliced in; DEG, differentially expressed gene; WGCNA, weighted gene co-expression network analysis.

The data analysis strategy of this research is shown in Figure 1. To procure relevant datasets, we conducted a search within the Gene Expression Omnibus (GEO) database (https://www.ncbi.nlm.nih.gov/geo/) using the search terms “dilated cardiomyopathy” and/or “DCM.” Datasets were selected based on the following criteria: (1) the study organism was *Homo sapiens*; (2) the study type was “expression profiling by array”; (3) each dataset comprised microarray data for both dilated cardiomyopathy (DCM) and healthy donor (HD) samples, with a minimum of three heart samples included; and (4) raw data were accessible for independent analysis. Ultimately, three datasets met these inclusion criteria: GSE79962 (9 DCM and 11 healthy donor samples) (Matkovich et al., 2017), GSE42955 (12 DCM and 5 healthy donor samples) (Molina-Navarro et al., 2013), and GSE3585 (7 DCM and 5 healthy donor samples) (Barth et al., 2006). GSE5406, generated at an independent center, was used for primary external validation and included 86 idiopathic DCM and 16 nonfailing left-ventricular samples. The dataset-specific batch effects of three datasets were eliminated and normalized using the ComBat function of the sva package (Leek et al., 2012).

### Identification of splicing factor genes in dilated cardiomyopathy

The splicing factor genes were accessed from the SpliceAid-F database (Giuliet et al., 2013). The circlize R package was employed to visualize the chromosomal locations of the identified splicing factor genes. The identified splicing factor genes were analyzed using the STRING database (https://string-db.org/) to construct a protein-protein interaction (PPI) network, thereby investigating their interaction relationships. Subsequently, a Wilcoxon test was used to analyzed the disparities in splicing factor expression levels between the DCM group and normal controls.

### Correlation analysis between splicing factor genes and immune features in DCM

To analyze the immune features in dilated cardiomyopathy, the ssGSEA was employed to explore immune infiltration, investigating diverse levels of pertinent immune traits (including 15 immune cell types and 13 immune-related functions) (Bindea et al., 2013) within the DCM patients and healthy donors. Meanwhile, the Gaussian-based method was utilized to compute the pathway enrichment scores for each sample, enabling a quantitative evaluation of the pathways. In addition, Spearman’s correlation analysis evaluated the correlation between splicing factor genes and the immune microenvironment, including immunocyte infiltration and pathway activation. The correlation heatmaps were generated to visualize associations between splicing factor genes and immune traits in DCM.

### Consensus clustering analysis

The “ConsensuClusterPlus' package (version 1.72.0) (Wilkerson and Hayes, 2010), which is based on unsupervised clustering algorithm was used to conduct consensus clustering analysis. To bolster the robustness and dependability of the clustering outcomes, the entire analytical procedure was replicated a thousand times. The parameters were configured as follows: item resampling, with the proportion of items to sample at 80%; gene resampling, wherein the proportion of features to sample was also 80%; a maximum quantified k, signifying the maximum clustering number to access, set at 9; resampling with the number of subsamples amounting to 1,000; the agglomerative hierarchical clustering algorithm designated as “hc” (hclust); while the distance metric defined as “pearson” (1 - Pearson correlation). Subsequently, the SFs-based subtype classification was validated by principal component analyses (PCA) with “factoextra” package.

### DEGs and functional enrichment analysis of two DCM clusters

To identify differentially expressed genes (DEGs) across DCM clusters, we employed the empirical Bayes approach using the “limma” package in R. DEGs were defined as genes exhibiting a fold change greater than 1.2 and an adjusted p-value less than 0.05. Downstream analysis involved gene enrichment using the “ClusterProfiler” Bioconductor package, focusing on Gene Ontology (GO) functional categories (biological process, cellular component, and molecular function) and Kyoto Encyclopedia of Genes and Genomes (KEGG) pathways.

### Identification of splicing factors-related hub genes in DCM

We performed Weighted Gene Co-expression Network Analysis (WGCNA) (Langfelder and Horvath, 2008) to identify genes correlating with disease activity in DCM. The analysis utilized gene expression data from DCM patient subgroups. To ensure data heterogeneity suitable for WGCNA, genes were pre-filtered based on the top 25% of median absolute deviation (MAD) across all samples. Only samples meeting pre-defined inclusion criteria were incorporated into downstream analyses. The analysis began with the construction of an adjacency matrix, which was then converted into a topological overlap matrix (TOM) using a selected soft-thresholding power β. Gene modules were identified through hierarchical clustering of the TOM dissimilarity matrix using the dynamic tree cut algorithm. Modules exhibiting high correlation between their eigengenes (correlation >0.8) were subsequently merged. Finally, module membership (MM) and gene significance (GS) were computed to identify the co-expressed genes most relevant to HF disease activity within target modules.

### Screening characteristic splicing factor genes via three machine learning methods in dilated cardiomyopathy

In this study, three machine learning methods were employed, namely, Random Forest, Least Absolute Shrinkage and Selection Operator Regression (LASSO), and Support Vector Machine - Recursive Feature Elimination (SVM-RFE) for the aim to screen the characteristic splicing factors. The Random Forest model was implemented using the “randomForest” R package. The number of trees was set to 1,000, and the number of variables randomly sampled at each split was set to the square root of the total number of features. The fundamental principle of LASSO regression involves identifying variables by iteratively searching for the λ value that minimizes classification error. This method is mainly utilized for screening feature variables and constructing the optimal classification model (Wang et al., 2019; Sanz et al., 2018). Specifically, the “glmnet” R package (Friedman et al., 2010) was employed to implement the LASSO algorithm, and the penalty parameter λ was set through ten-fold cross-validation. SVM-RFE is a machine learning approach built upon the support vector machine. It identifies the optimal variables by screening the feature vectors generated by the support vector machine. Additionally, the optimal variables were screened by executing the SVM-RFE algorithm with ten-fold cross-validation. The identification of characteristic splicing factor genes was determined by integrating the outputs of the Random Forest, LASSO, and SVM-RFE methodologies. To reduce optimistic bias, model performance was evaluated using repeated nested stratified cross-validation, with 5-fold outer cross-validation repeated 20 times. Within each outer training set, feature selection was repeated independently using LASSO, random forest, and SVM-RFE. Features selected by at least two algorithms were used to fit a ridge logistic regression model, which was then applied to the corresponding held-out fold. Hyperparameters and feature-selection settings were optimized exclusively within the outer training set using inner cross-validation. The predictions generated for each sample across the repeated outer folds were averaged to construct the out-of-fold ROC curve.

### Differential alternative splicing analysis

Differential alternative splicing events were detected using rMATS (v4.2.0) based on junction-count (JC) output. The analysis was performed in single-end mode with an unstranded library setting (fr-unstranded) and a read length of 51 bp. Five classes of AS events were evaluated, including skipped exon (SE), mutually exclusive exon (MXE), retained intron (RI), alternative 5′splice site (A5SS), and alternative 3′splice site (A3SS). Events with FDR <0.05 and |IncLevelDifference| ≥ 0.1 were considered significant. rMATS reports AS changes at the event level; therefore, multiple events from the same gene were retained as distinct AS events and were not collapsed into gene-level counts. The inclusion-level difference was calculated as PSI_DCM − PSI_Control. Significant DAS events were summarized according to AS type and inclusion direction, and event-level PSI matrices were used for principal component analysis and heatmap visualization.

### Functional enrichment and visualization of representative DAS events

Genes harboring significant DAS events were subjected to functional enrichment analysis based on Gene Ontology, KEGG, and Reactome terms (Rothfels et al., 2023) relevant to DCM pathobiology. Representative DAS events were selected according to statistical significance, PSI change magnitude, junction-read support, event interpretability, and biological relevance to cardiac structure or function. Group-level splice-junction usage for selected events was visualized using rMATS-derived event-level junction counts, and PSI distributions were compared between DCM and control samples. These junction-count schematics were used to provide event-level support from rMATS output and were not interpreted as BAM-derived sashimi plots.

### Construction of the characteristic SF-AS network

To link characteristic splicing factors with DCM-associated AS remodeling, expression levels of *MBNL1*, *NOVA1*, *RBM25*, *RBM5*, and *SF1* were correlated with PSI values of the top 300 DAS events ranked by FDR and absolute inclusion-level difference. SF expression was quantified as log2(CPM +1) from gene-level count data, and PSI values were extracted from rMATS inclusion-level estimates. Spearman correlation analysis was performed for each SF–AS event pair, followed by Benjamini–Hochberg correction for multiple testing. Associations with adjusted FDR <0.05 were retained, and those with |R| > 0.5 were defined as strong correlations. The resulting network integrated characteristic SFs, key DAS events, and corresponding functional modules to illustrate putative regulatory relationships underlying DCM-associated splicing remodeling.

### Statistical analyses

All statistical analyses were performed using R software (version 4.4.2). Continuous variables between two groups were compared using the Wilcoxon rank-sum test. For multiple comparisons, p-values were adjusted using the false discovery rate (FDR) method. For standard statistical comparisons, a two-sided p-value <0.05 was considered statistically significant. Correlations between variables were evaluated using either Pearson or Spearman correlation analysis, depending on the distributional characteristics of the data. The diagnostic performance of the characteristic splicing factors was evaluated via receiver operating characteristic (ROC) curve analysis, and the area under the curve (AUC) was calculated to quantify discriminative ability.

## Results

### Global landscape of alternative splicing alterations in DCM

**FIGURE 2:**
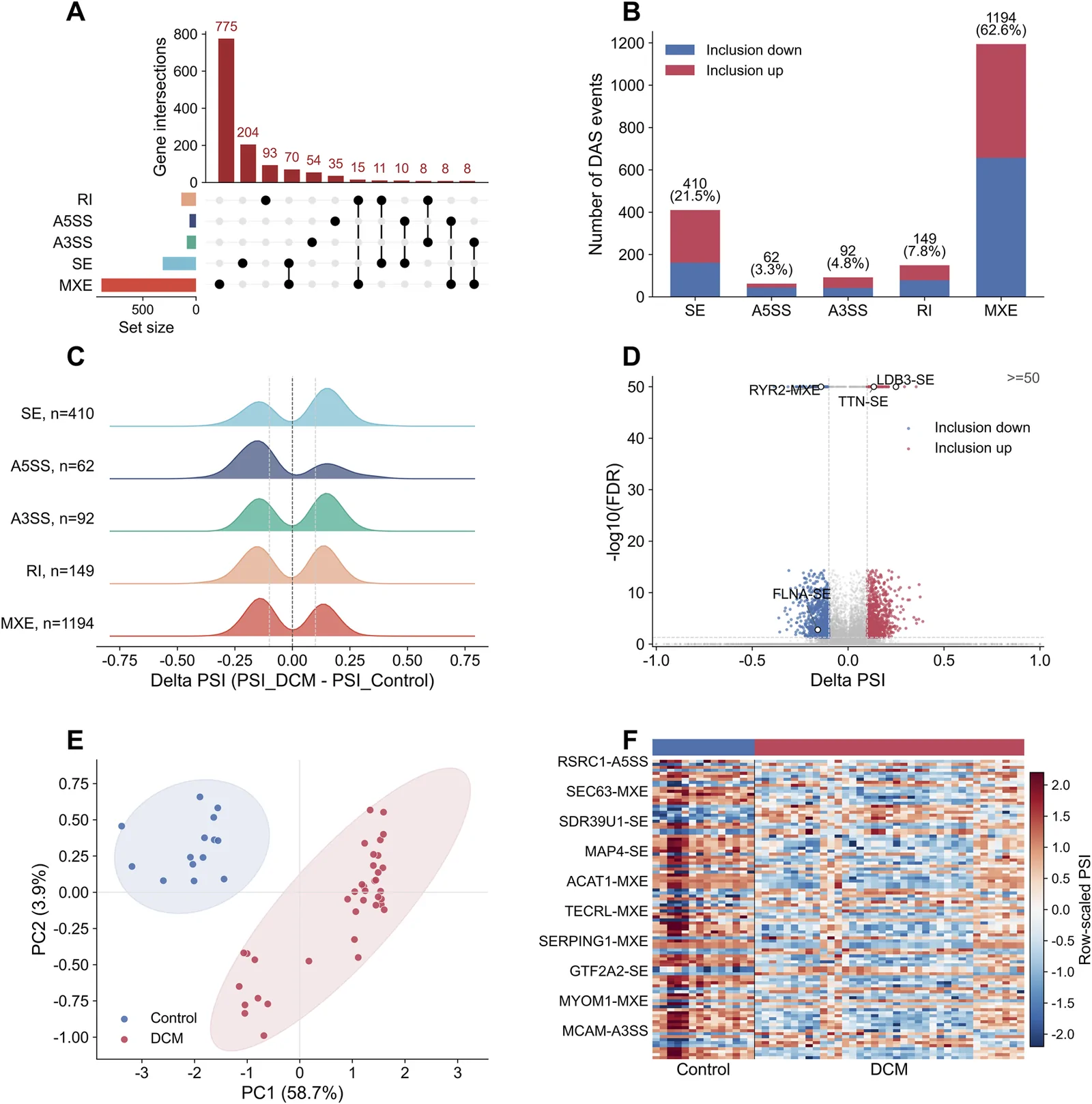
Global landscape of alternative splicing alterations in DCM. **(A)** UpSet plot showing the overlap of genes harboring different types of significant DAS events, including skipped exon (SE), alternative 5′splice site (A5SS), alternative 3′splice site (A3SS), retained intron (RI), and mutually exclusive exon (MXE). **(B)** Stacked bar plot showing the number of DAS events across different AS types. Blue and red bars indicate inclusion-down and inclusion-up events in DCM, respectively. **(C)** Ridge density plots showing the distribution of ΔPSI values across AS types. ΔPSI was calculated as PSI_DCM − PSI_Control. **(D)** Volcano plot showing DAS events between DCM and control samples. Red dots indicate inclusion-up events in DCM, blue dots indicate inclusion-down events in DCM, and grey dots indicate non-significant or filtered events. Dashed lines indicate the thresholds used for DAS identification. Values with very high -log10(FDR) were capped for visualization if applicable. **(E)** PCA based on PSI profiles of significant DAS events, showing the separation between DCM and control samples. **(F)** Heatmap showing row-scaled PSI values of representative DAS events across DCM and control samples.

The data analysis strategy of this research is shown in Figure 1. To systematically characterize alternative splicing alterations in DCM, we first analyzed differential alternative splicing (DAS) events between DCM and control samples. Significant DAS events were detected across multiple AS types, including skipped exon (SE), alternative 5′splice site (A5SS), alternative 3′splice site (A3SS), retained intron (RI), and mutually exclusive exon (MXE). Using FDR <0.05 and |IncLevelDifference| ≥ 0.1, we identified 1,907 differential AS events involving 1,315 genes, including 410 SE, 1,194 MXE, 92 A3SS, 62 A5SS, and 149 RI events. Among these, MXE represented the most abundant AS category, followed by SE (Figures 2A,B). Among these, MXE represented the most abundant AS category, followed by SE, suggesting extensive exon usage remodeling in DCM (Figures 2A,B). The distribution of ΔPSI values showed both inclusion-up and inclusion-down events across AS types, indicating bidirectional splicing alterations rather than a uniform shift in exon inclusion (Figure 2C). Volcano plot analysis further confirmed widespread DAS events with significant PSI changes between DCM and controls (Figure 2D). In addition, PCA based on PSI profiles of significant DAS events separated DCM samples from controls, supporting the presence of disease-associated AS remodeling (Figure 2E). A heatmap of representative DAS events further demonstrated distinct PSI patterns between the two groups (Figure 2F). Together, these results reveal a global remodeling of alternative splicing in DCM.

### Functional relevance and representative DAS events in DCM

**FIGURE 3:**
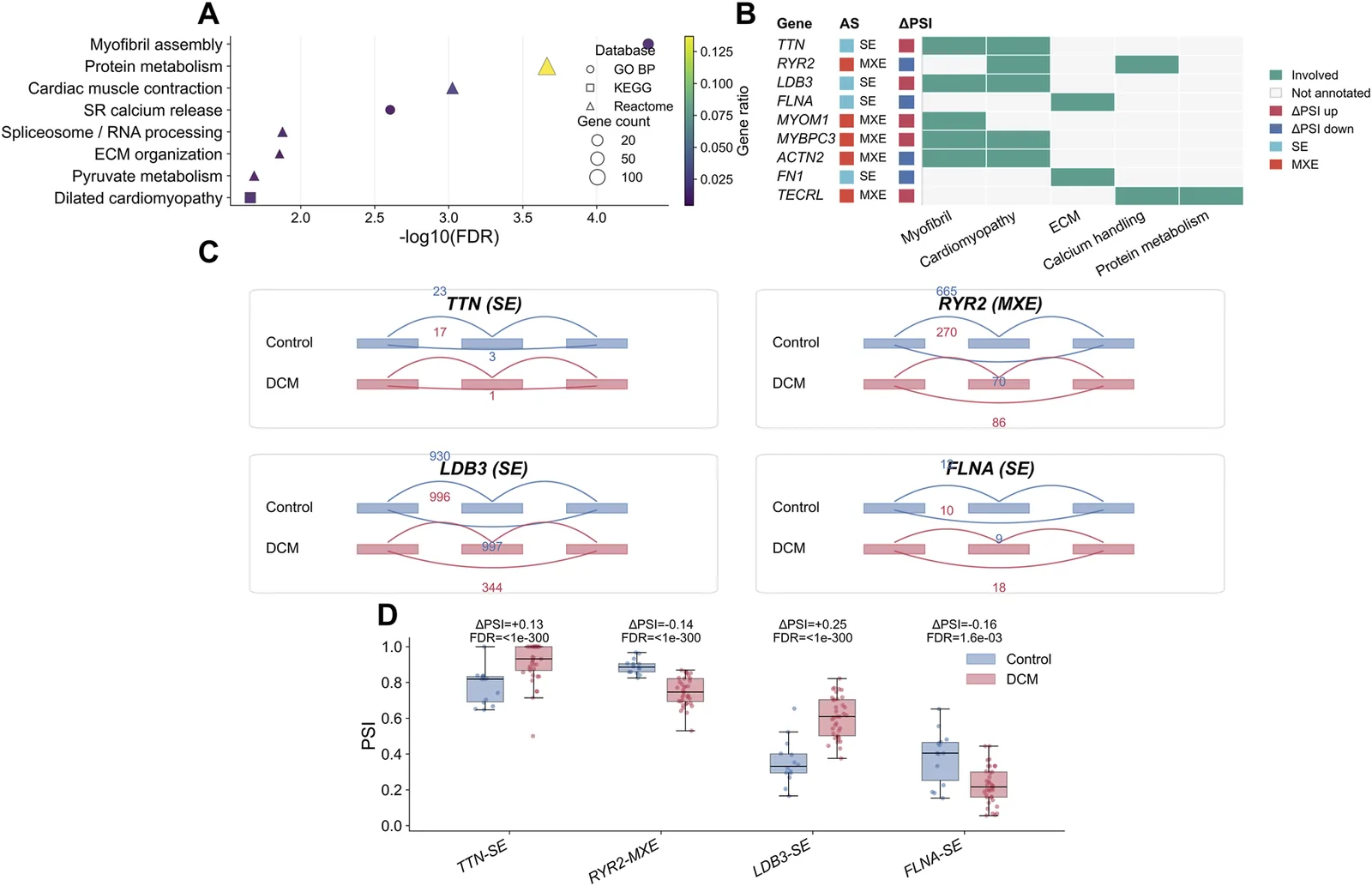
Functional relevance and representative splicing events of DCM-associated DAS genes. **(A)** Functional enrichment dot plot of genes harboring significant DAS events. Dot size represents gene count, color indicates -log10(FDR), and dot shape indicates the database source. **(B)** DAS gene-functional module matrix showing the relationship between representative DAS genes and DCM-relevant functional modules. AS type and ΔPSI direction are annotated on the left. Green blocks indicate functional module involvement. **(C)** Representative sashimi-like junction-count schematics for selected high-confidence DAS events in *TTN*, *RYR*2, *LDB3*, and *FLNA*. Control and DCM groups are shown separately, with junction counts, ΔPSI, and FDR values indicated for each event. **(D)** PSI distribution of representative DAS events between control and DCM samples. ΔPSI was calculated as PSI_DCM–PSI_Control.

We next investigated the biological relevance of genes harboring significant DAS events. Functional enrichment analysis showed that DAS genes were enriched in DCM-relevant processes, including SR calcium release, cardiac muscle contraction, myofibril assembly, ECM organization, protein metabolism, pyruvate metabolism, dilated cardiomyopathy, and spliceosome/RNA processing pathways (Figure 3A). A DAS gene–functional module matrix further linked representative DAS genes to key functional modules, including myofibril organization, cardiomyopathy-related processes, ECM remodeling, calcium handling, and protein metabolism (Figure 3B). To provide junction-level evidence, we selected representative high-confidence DAS events in *TTN*, *RYR2*, *LDB3*, and *FLNA* based on statistical significance, junction-read support, biological relevance, and clear event structure. Sashimi-like junction-count schematics showed distinct splice-junction usage between DCM and control samples for these events (Figure 3C). The raw inclusion-junction and skipping-junction counts for the representative events shown in Figure 3C are provided in Supplementary Data 5. Labels in Figure 3C indicate rMATS inclusion- and skipping-junction counts; extremely small P values or FDR values reported for these events reflect numerical underflow. Consistently, PSI distribution analysis confirmed significant PSI shifts in TTN-SE, RYR2-MXE, LDB3-SE, and FLNA-SE (Figure 3D).

### Landscape of splicing factors in dilate“d cardiomyopathy

**FIGURE 4:**
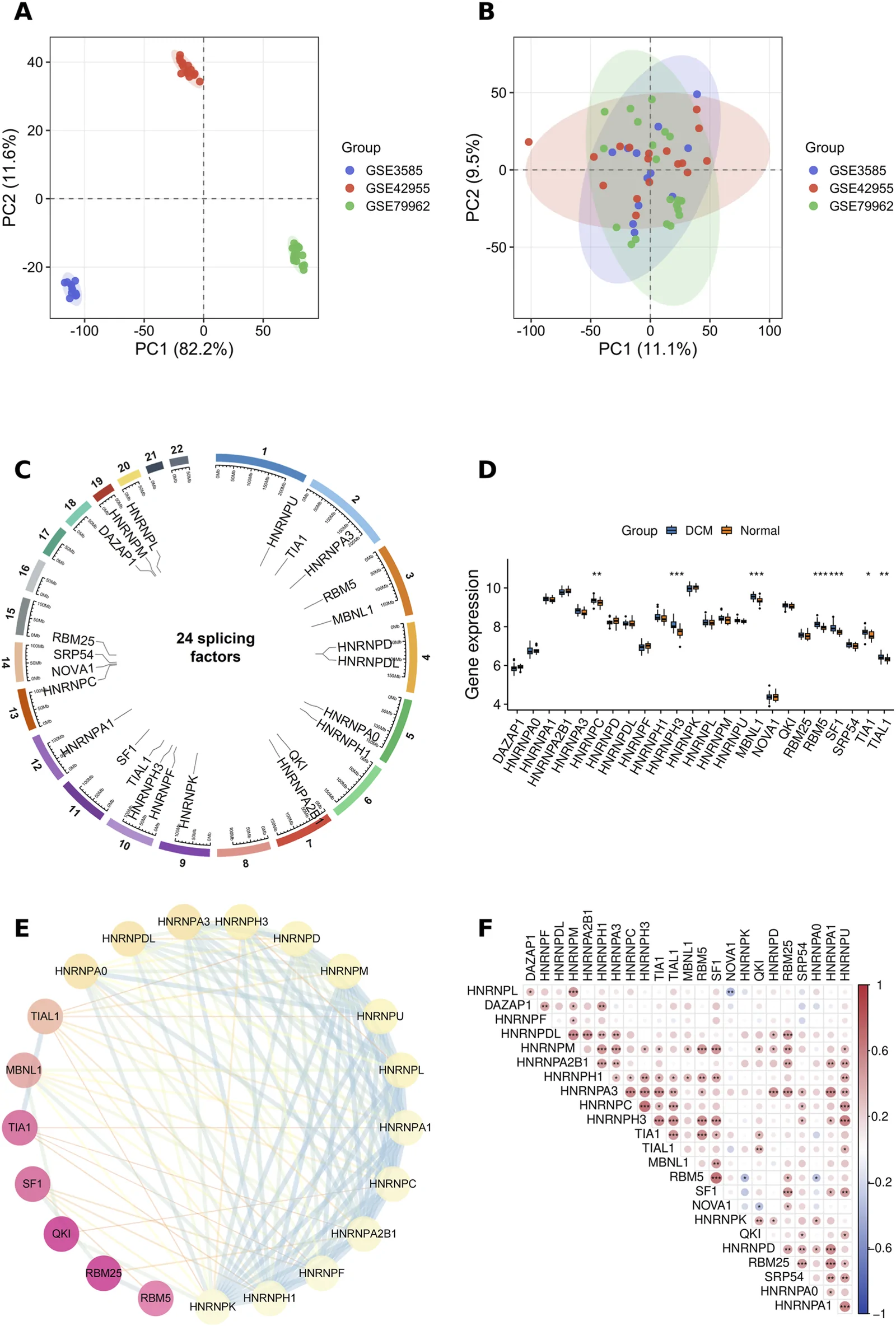
Systemic Profiling of splicing factors in Dilated Cardiomyopathy. **(A)** PCA visualization of three GEO datasets (GSE3585/GSE42955/GSE79962) pre-normalization; **(B)** Characterizing post-normalization dataset distribution through PCA dimensionality reduction; **(C)** Circular genome visualization of splicing factor chromosomal localization; **(D)** Differential splicing factor dysregulation: Heatmap quantification across DCM versus control cohorts; **(E)** STRING-based PPI network topology among the 24 splicing factors; **(F)** Correlations among the 24 splicing factors for DCM and normal samples (*p < 0.05, **p < 0.01, ***p < 0.001).

The efficacy of batch correction was validated through principal component analysis (PCA), with pre- and post-normalization distributions visualized in Figures 4A,B, respectively. In order to investigate the intricate splicing processes mediated by splicing factors in dilated cardiomyopathy, we initially retrieved 24 pertinent splicing factors from the SpliceAid-F database. The chromosomal locations of the detected splicing factors are presented in Figure 4C. Subsequently, we analyzed the expression features of the 24 splicing factors in both dilated cardiomyopathy and normal samples. The normalized expression matrix of the 24 splicing factors is provided in Supplementary Data 1. The results revealed that, as compared to normal samples, the expression of seven splicing factors (*MBNL1*, *HNRNPH3*, *RBM5*, *SF1*, *HNRNPC*, TIA1, *TIAL1*) was significantly elevated in dilated cardiomyopathy samples (Figure 4D). Analysis of the protein-protein interaction network constructed via the STRING database demonstrated strong associations among the 24 splicing factors studied, suggesting they may perform their biological functions as a complex (Figure 4E). Our correlation analysis revealed significant positive associations among splicing factors, establishing a foundation for subsequent clustering studies. Notably, *RBM5* and *SF1* exhibited the strongest co-expression relationship among regulatory factors, implying their potential functional synergy in shared biological processes (Figure 4F).

### Splicing factors are associated with immune characteristics of DCM

**FIGURE 5:**
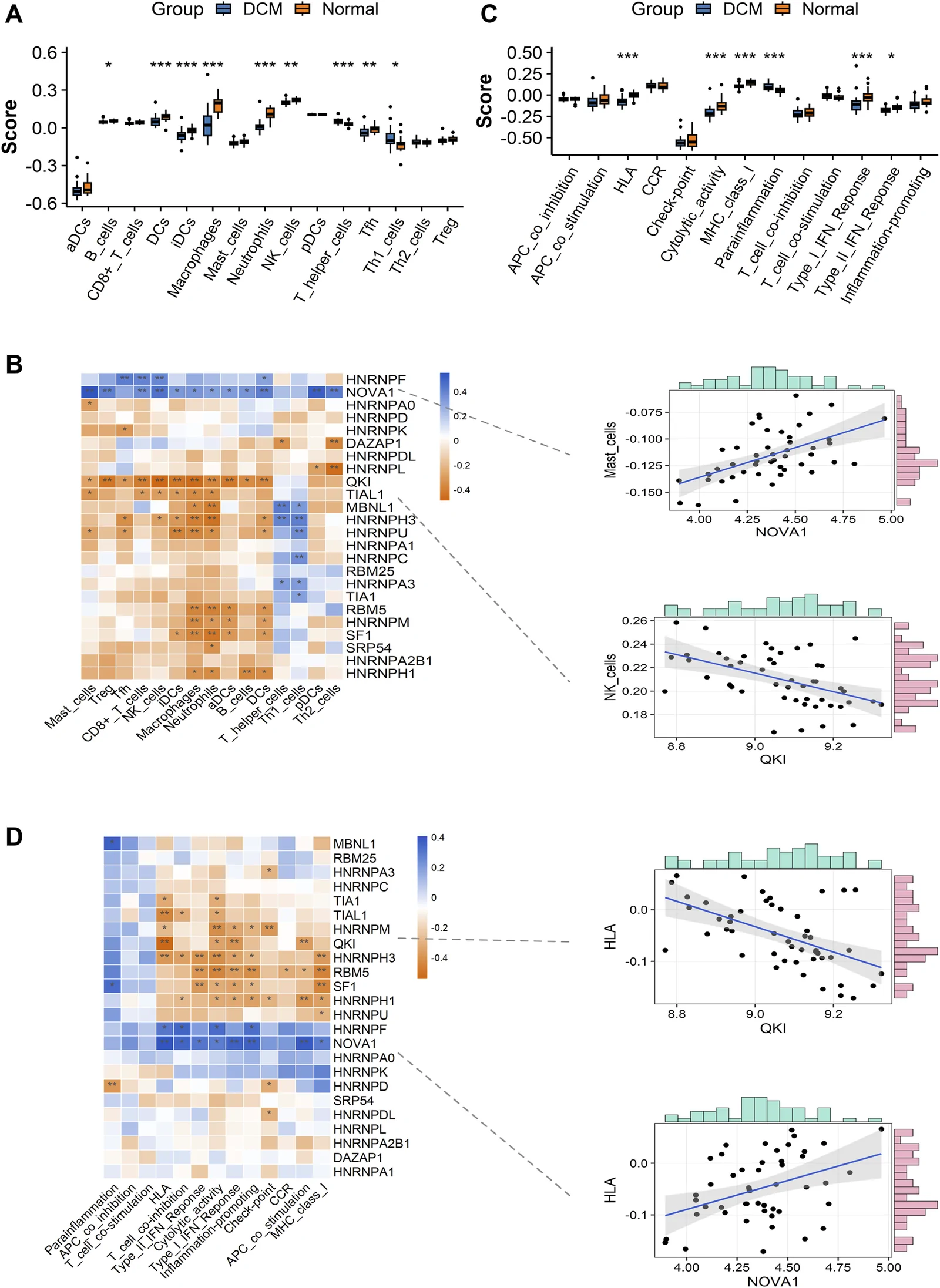
Splicing factors are associated with immune characteristics of DCM. **(A)** Comparison of ssGSEA-derived infiltration scores for 15 immune cell types between DCM and normal samples (*p < 0.05, **p < 0.01, and ***p < 0.001). **(B)** Comparison of ssGSEA-derived scores for 13 immune-related functions between DCM and normal samples. **(C)** Spearman’s rank correlation matrix between the expression of 24 splicing factors and 15 immune-cell infiltration scores within DCM samples. **(D)** Spearman’s rank correlation matrix between the expression of 24 splicing factors and 13 immune-related functional scores within DCM samples.

To systematically investigate the interplay between splicing factors and immunological profiles in dilated cardiomyopathy (DCM), we conducted integrated correlation analyses using ssGSEA-derived immune metrics. Our computational framework quantified infiltration levels of 15 immune cells and activity scores of 13 immune functional pathways across patient cohorts. Comparative analysis revealed distinct immune landscape alterations in DCM, with nine immune cell populations demonstrating significant infiltration differences compared to controls (Figure 5A). Immune cell enrichment scores calculated by ssGSEA are shown in Supplementary Data 3. Immune functional characterization uncovered six significantly modulated pathways in DCM pathogenesis (Figure 5B), including antigen presentation machinery (HLA, MHC class I), cytolytic activity, inflammatory signaling (Parainflammation), and interferon responses (types I/II IFN). Immune functional activity scores are provided in Supplementary Data 4. We assessed the associations between splicing-factor expression and immune features exclusively within DCM samples. The DCM-only correlation heatmap revealed broad associations between dysregulated splicing factors and immune-cell enrichment patterns, with several factors, including *NOVA1*, *QKI*, *HNRNPU*, *DAZAP1* and *HNRNPF*, showing prominent correlations with specific immune-cell subsets (Figure 5C). Representative associations included a positive correlation between *NOVA1* expression and Treg enrichment (r = 0.50, p < 0.01) and a negative correlation between *QKI* expression and Treg enrichment (r = −0.65, p < 0.01). For immune functional scores, the DCM-only correlation heatmap showed that splicing factors were differentially associated with immune functional programs, particularly antigen-presentation- and inflammatory-response-related activities (Figure 5D). Among these associations, *QKI* expression was inversely correlated with HLA activity (r = −0.75, <0.01). Together, these analyses suggest that dysregulated splicing factors are associated with immune-cell and immune-function heterogeneity in DCM.

### Screening characteristic splicing factors through three machine learning algorithms

**FIGURE 6:**
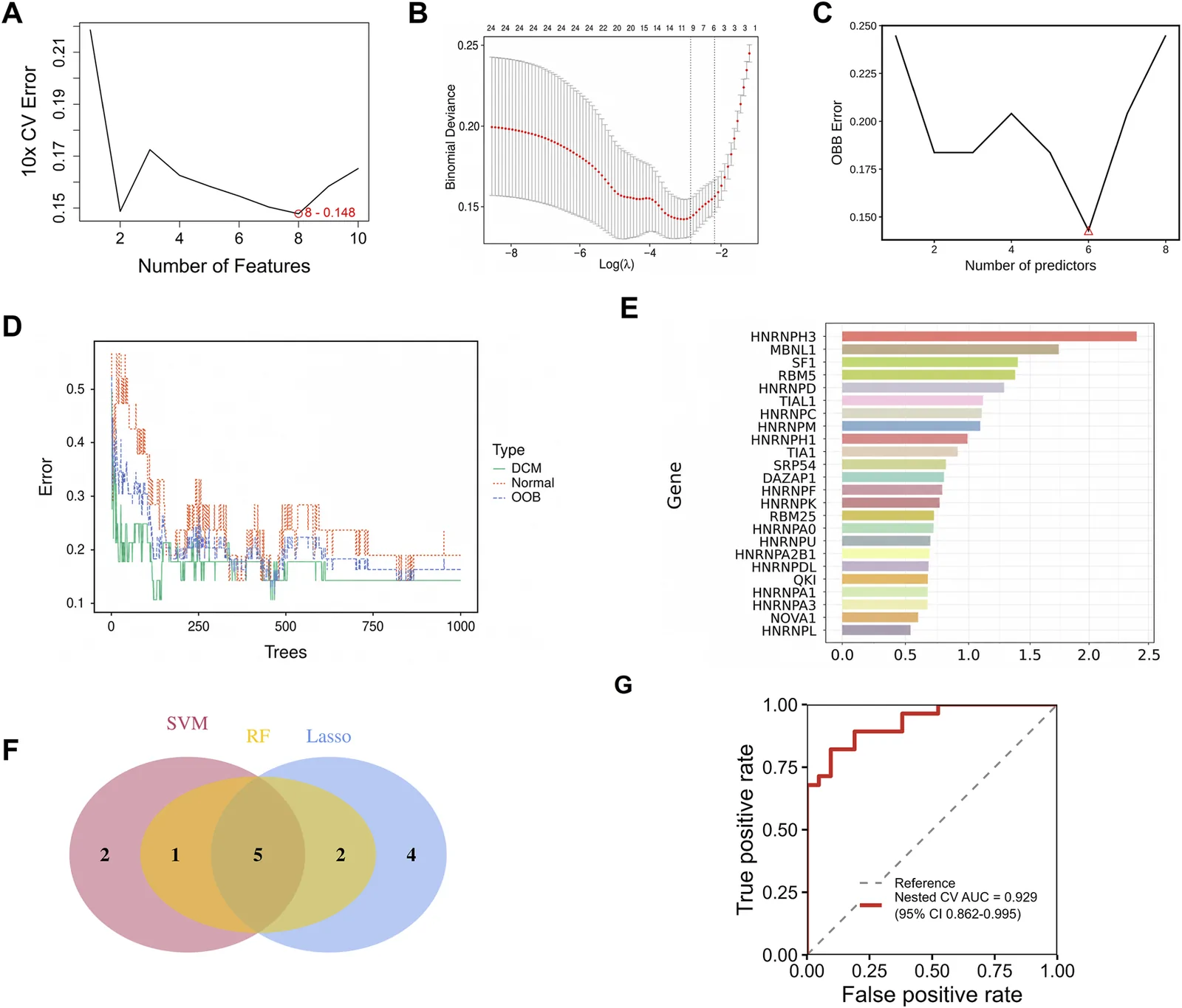
Screening characteristic splicing factors by three machine learning algorithms. **(A)** Search for the optimal value of mtry for SVM-REF model; **(B)** Least absolute shrinkage and selection operator (LASSO) regression algorithm to identify characteristic markers; **(C)** Out-of-bag (OOB) error rate of the random forest (RF) model; **(D)** Search for the optimal value of mtry for RF model; **(E)** Variable importance plot for the RF model; **(F)** Venn diagram illustrating the intersection of genes identified by SVM-RF, Lasso, and RF algorithm; **(G)** Out-of-fold receiver operating characteristic curve of the consensus classifier generated using repeated nested stratified cross-validation. The classifier achieved an AUC of 0.929 (95% CI, 0.862–0.995).

To establish a splicing factor-based diagnostic signature for dilated cardiomyopathy (DCM) prediction, we implemented a multi-algorithm approach incorporating three distinct machine learning methodologies: LASSO regression, Random Forest, and SVM-RFE feature selection. Initial SVM-RFE analysis identified an optimal feature subset comprising eight candidate biomarkers, demonstrating the lowest 10-fold cross-validation error rate (Figure 6A). Subsequent application of regularization techniques through LASSO regression and Random Forest algorithms revealed comparative coefficient profiles, with the latter employing Gini index reduction for feature prioritization (Figures 6B–D). Critical analysis of feature importance rankings identified *HNRNPH3*, *MBNL1*, *HNRNPD*, *RBM5*, and *SF1* as top discriminative features through Random Forest evaluation (Figure 6E). Intersectional analysis of results from all three methodologies (SVM-RFE, LASSO, and Random Forest) converged to identify five consensus biomarkers: *MBNL1*, *NOVA1*, *RBM25*, *RBM5*, and *SF1* (Figure 6F). Repeated nested stratified cross-validation yielded an out-of-fold AUC of 0.929 (95% CI, 0.862–0.995) for the consensus classifier in the merged discovery cohort (Figure 6G).

### Characteristic splicing factors are associated with DCM-related alternative splicing events

**FIGURE 7:**
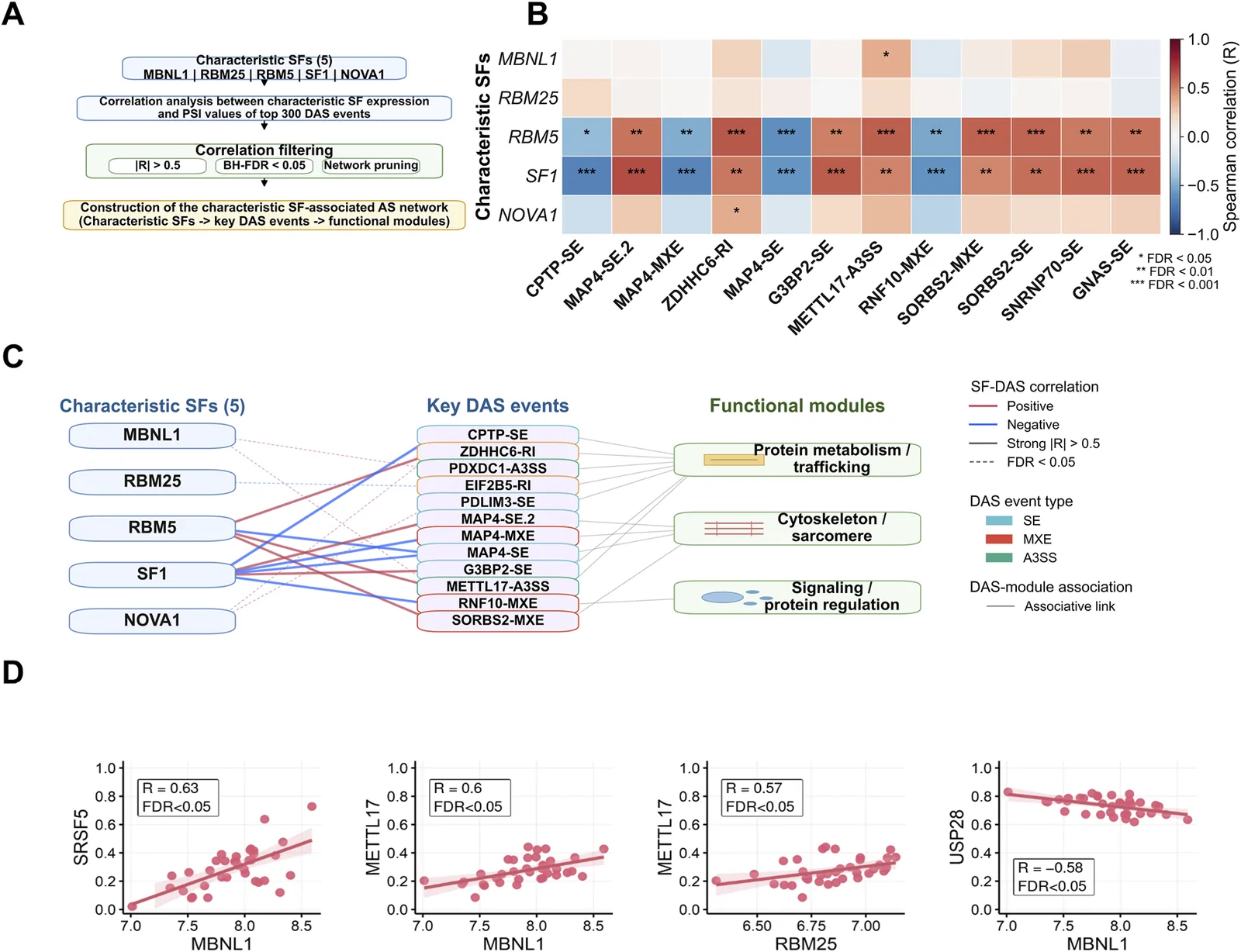
Characteristic SF-AS network in DCM. **(A)** Workflow for construction of the characteristic SF–AS network. Expression levels of five characteristic SFs were correlated with PSI values of the top 300 DAS events identified by rMATS, followed by correlation filtering and network pruning. **(B)** Correlation heatmap showing the associations between characteristic SF expression and representative DAS events. Color indicates the Spearman correlation coefficient (R), and asterisks denote significance levels. **(C)** Characteristic SF-AS network linking five characteristic SFs to key DAS events and downstream functional modules. Solid red and blue edges indicate strong positive and negative correlations, respectively (|R| > 0.5), whereas dashed edges indicate weaker but significant associations (FDR <0.05). Grey lines represent associations between DAS events and functional modules. Different border colors of DAS-event nodes indicate AS event types. **(D)** Representative scatter plots showing correlations between characteristic SF expression and PSI values of selected DAS events in DCM samples. Spearman correlation coefficients and FDR values are shown in each panel.

To further connect upstream splicing regulators with DCM-associated alternative splicing remodeling, we constructed a characteristic splicing factor–alternative splicing (SF-AS) network using five characteristic SFs (*MBNL1*, *RBM25*, *RBM5*, *SF1*, and *NOVA1*). Correlation analysis was performed between characteristic SF expression and PSI values of the top 300 DAS events identified by rMATS, followed by filtering using BH-FDR <0.05 and network pruning (Figure 7A). The correlation heatmap revealed distinct association patterns between characteristic SFs and representative DAS events, with *RBM5* and *SF1* showing the most prominent and extensive correlations, whereas *MBNL1*, *RBM25*, and *NOVA1* displayed fewer but still retained significant associations (Figure 7B). Based on these results, we established a characteristic SF-associated AS network linking all five SFs to key DAS events and downstream functional modules (Figure 7C). In this network, strong SF-AS correlations (|R| > 0.5) were represented by solid edges, whereas weaker but significant associations (FDR <0.001) were shown as dashed edges. The identified key DAS events were mainly associated with protein metabolism/trafficking, cytoskeleton/sarcomere organization, and signaling/protein regulation. Notably, *SF1* and *RBM5* occupied relatively central positions in the network, suggesting that they may play major roles in coordinating DCM-related splicing alterations. Representative SF-AS pairs showed the strongest nominal within-DCM associations. For example, MBNL1 expression was positively correlated with SRSF5-A3SS and METTL17-A3SS PSI, RBM25 expression was positively correlated with METTL17-A3SS PSI, whereas MBNL1 expression showed a negative association with USP28-MXE PSI (Figure 7D). Collectively, these findings suggest that characteristic SFs are associated with DCM-related transcriptomic remodeling and functionally relevant AS events, although the underlying regulatory relationships require further experimental validation.

### Unsupervised clustering of DCM samples based on splicing factors

**FIGURE 8:**
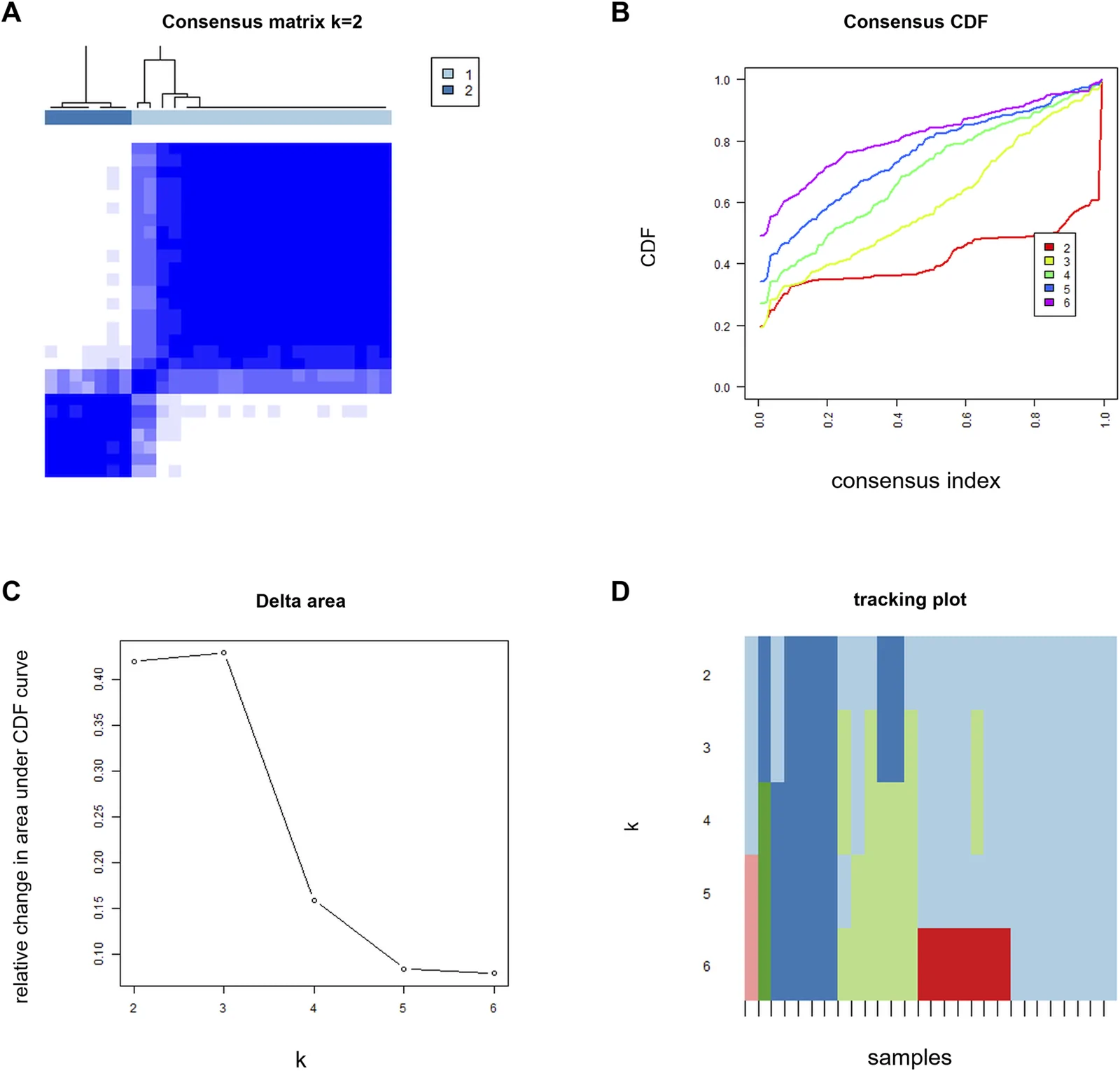
Construction of two splicing factors-based subtypes for DCM. **(A)** Consensus matrix heatmap defining two subtypes (k = 2) and their correlation area; **(B)** Consensus CDF curves at k = 2–6. **(C)** The delta area plot; **(D)** The tracking plot.

Consensus clustering analyses were conducted to stratify dilated cardiomyopathy patients into distinct gene clusters, utilizing the expression values of 24 splicing factors. The resulting consensus matrix heatmap for k = 2 (Figures 8A,B) clearly demonstrates that DCM samples can be effectively partitioned into two SF-based clusters: Cluster A (n = 21) and Cluster B (n = 7). The delta area plot (Figure 8C) reveals a minimal increase in the area under the cumulative distribution function (CDF) curve when increasing k from 2 to 4, thereby providing robust support for k = 2 as the optimal number of clusters. The tracking plot illustrated changes in sample assignments across different k values (Figure 8D). Based on these comprehensive analyses, two distinct SF-based clusters were ultimately defined within the dilated cardiomyopathy cohort, offering insights into potential molecular subtypes of the disease.

### Immune characteristics and pathways in two distinct splicing factor clusters

**FIGURE 9:**
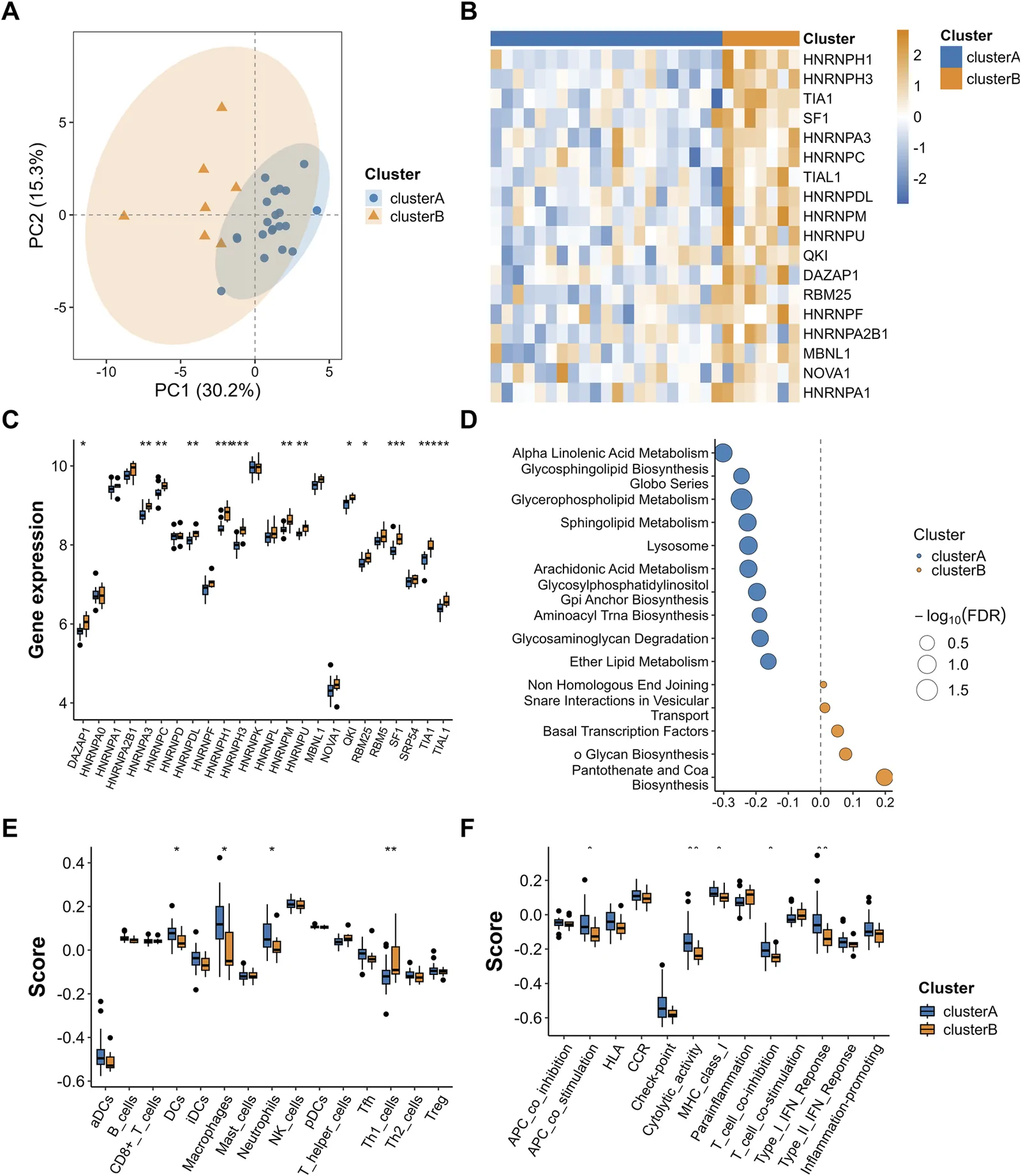
Immune characteristics and biological pathways in two DCM clusters. **(A)** Principal component analysis (PCA) presenting a notable difference in transcriptomes between the two clusters of DCM; **(B)** Heatmap of splicing genes between the two DCM clusters; **(C)** Boxplots quantify distinct expression profiles of the 24 splicing factors in two DCM clusters, *p < 0.05, **p < 0.01, ***p < 0.001 vs. clusterA; **(D)** GSVA for comparing biological pathways function between clusterA and cluster; **(E)** The infiltration scores of 15 cells infiltrations between two DCM clusters; **(F)** The infiltration scores of 13 immune-related functions between two DCM clusters.

Distinct expression profiles were identified between the two clusters, as revealed by principal component analysis (PCA). The accuracy of this classification was validated using PCA plots. As shown in Figures 9A,B, these analyses demonstrate the presence of two distinct types of DCM samples. Subsequently, we analyzed the expression patterns of the 24 splicing regulators in two distinct clusters. The results showed that, compared to cluster A, the expression of thirteen splicing factors (*DAZAP1*, HNRNPA1, *HNRNPD*L, *HNRNPH1*, *HNRNPM*, *HNRNPH3*, *HNRNPU*, *QKI*, *RBM25, SF1*, *HNRNPC*, TIA1, *TIAL1*) was significantly elevated in cluster B (Figure 9C). Gene-set variation analysis (GSVA) identified significant enrichments in specific pathways for cluster A compared to cluster B. Specifically, cluster A exhibited enhanced activity in several key biological processes, including alpha-linolenic acid metabolism, arachidonic acid metabolism, glycosphingolipid biosynthesis, glycerophospholipid metabolism, sphingolipid metabolism, lysosome, and glycosaminoglycan degradation (Figure 9D). To explore further insight into between the two SF clusters and immunological features in DCM, we performed the ssGSEA algorithm to assess differences in infiltrating immune cell abundance and activity levels of immune-related biological pathways between these clusters. As depicted in Figure 9E, cluster A demonstrated elevated levels of dendritic cells (DCs), macrophages, and neutrophils compared to cluster B. Furthermore, for immune-related functions, cluster A in DCM showed marked upregulation of cytolytic activity, Type I interferon response, and antigen-presenting cell (APC) costimulatory pathways, as illustrated in Figure 9F. These findings collectively indicate that the two distinct splicing factor clusters are associated with unique immune signatures and functional profiles. This suggests that RNA splicing regulators may play a critical role in shaping the immune microenvironment of DCM.

### Identification of DCM cluster-associated DEGs

**FIGURE 10:**
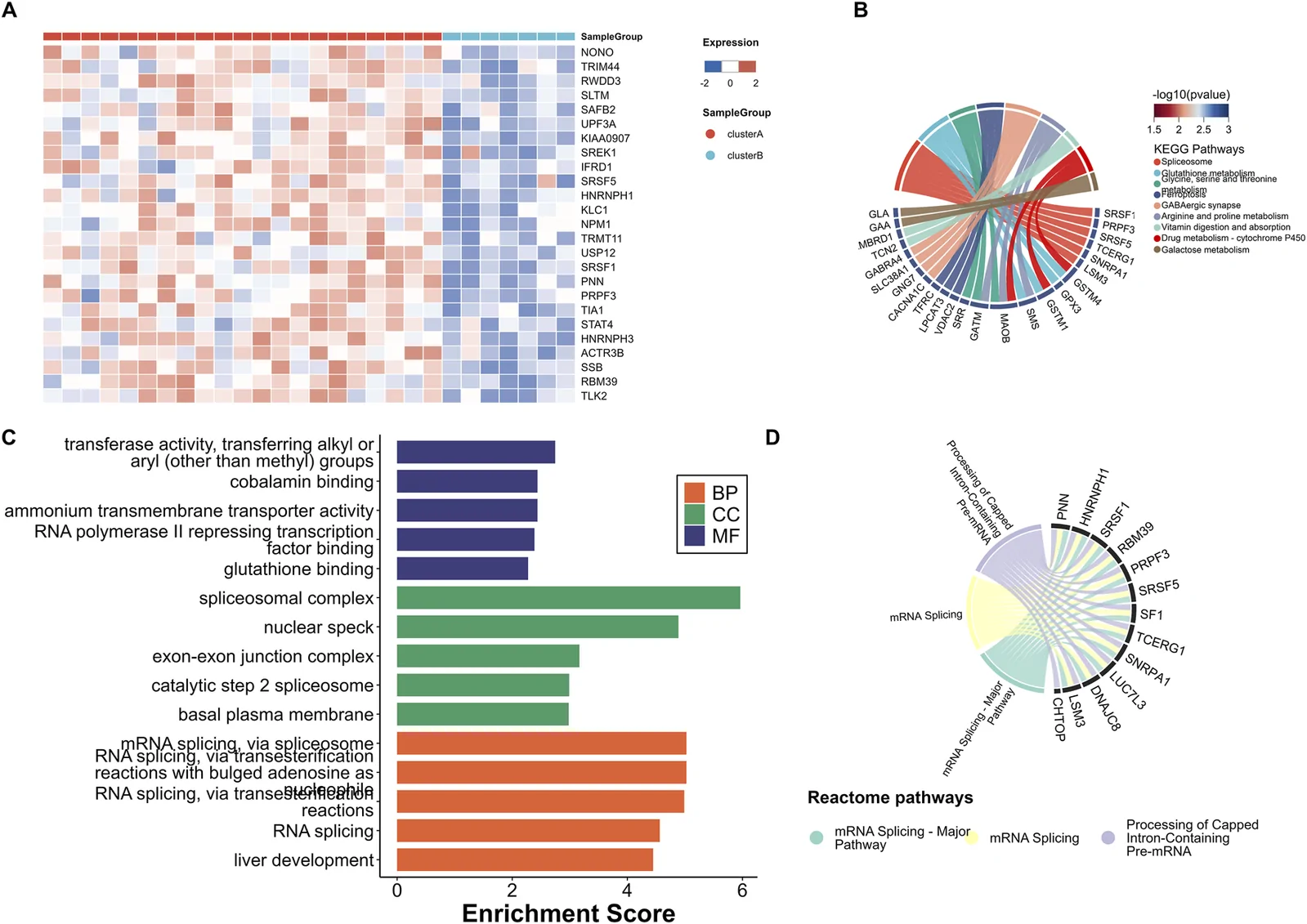
DEGs and pathway enrichment between two DCM clusters. **(A)** Heatmap of DEGs between two DCM clusters; **(B)** KEGG enrichment analysis of DEGs between two DCM clusters; **(C)** GO enrichment analysis of DEGs between two DCM clusters. BP, biological process, CC, cellular components, MF, molecular functions; **(D)** Reactome enrichment analysis of DEGs between two DCM clusters.

Using the limma package, we identified 181 differentially expressed genes (DEGs) between the two SF-defined DCM clusters based on an adjusted P value <0.05 and ∣log2FC∣>1.2. The heatmap demonstrated distinct gene-expression patterns between the two clusters (Figure 10A). Functional enrichment analyses based on Gene Ontology (GO), Kyoto Encyclopedia of Genes and Genomes (KEGG), and Reactome indicated that these DEGs were enriched in biological processes and pathways related to cardiac function, myocardial metabolism, and RNA processing (Figures 10B–D). Collectively, these results indicate that the SF-defined DCM clusters are associated with distinct RNA-processing, cardiac, and metabolic characteristics; however, enrichment analysis alone does not establish a causal role for alternative splicing regulation in myocardial substrate utilization or energy homeostasis.

### Identification of SFs-related hub genes in DCM

**FIGURE 11:**
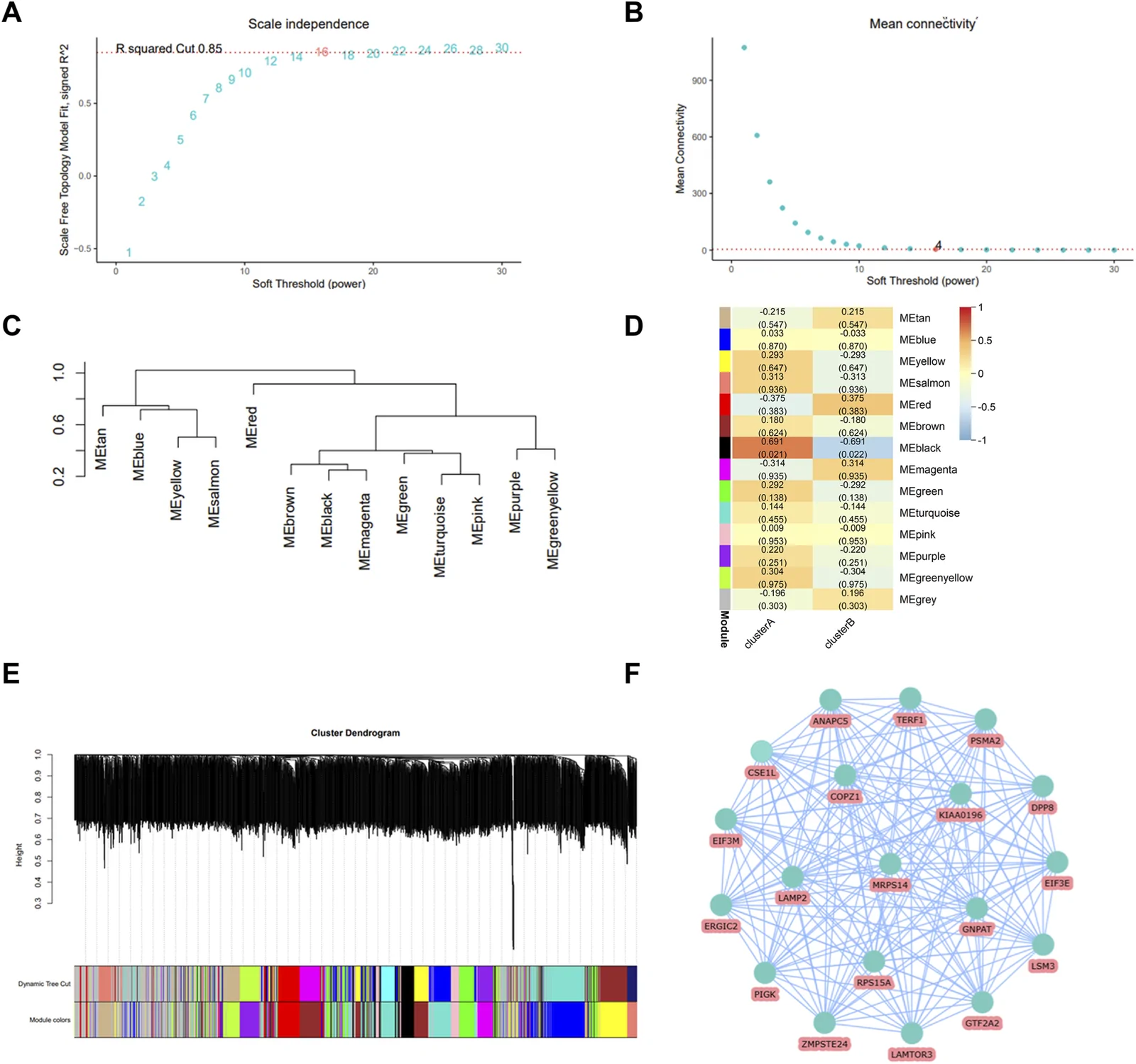
Construction of WGCNA network and identification of SFs-related hub genes in DCM. **(A)** Scale-free topology index analysis and mean connectivity of soft threshold power from 1 to 30. The red line displays the scale-free R^2^ (0.85); **(B)** The effect of soft threshold power on mean connectivity; **(C)** Clustering dendrogram of module eigengenes; **(D)** Heatmap of the correlation between the module eigengenes and DCM clusters; **(E)** Gene dendrogram clustered through average linkage hierarchical clustering and merged when the correlation of the modules is >0.8; **(F)** Protein–protein interaction (PPI) network of genes from the black module.

Weighted gene co-expression network analysis (WGCNA) depicts hierarchical clustering dendrogram in 28 DCM samples, as shown in the dendrogram and trait clustering. The scale-free topology fitting index (R^2^ = 0.85) confirmed optimal network connectivity at a soft threshold (β = 16) (Figures 11A,B). Subsequent module merging with a height cutoff of 0.2 and dynamic branch cutting identified 13 co-expression modules through hierarchical clustering (Figure 11C). Notably, the MEblack module demonstrated the strongest correlation with DCM cluster A (Pearson’s r = 0.691, p < 0.05) in the module-trait association heatmap, establishing its pivotal role in disease pathogenesis (Figures 11D,E). Subsequently, 110 genes in the MEblack module were used to construct the PPI network, and hub genes were selected by cytoHubba in Cytoscape software. Finally, six genes including *COPZ1*, *LAMP2*, *KIAA0196*, *MRPS14*, *RPS15A*, and *GNPAT* were identified (Figure 11F). Gene-module assignments derived from WGCNA are summarized in Supplementary Data 2.

### External verification of characteristic genes in dilated cardiomyopathy

**FIGURE 12:**
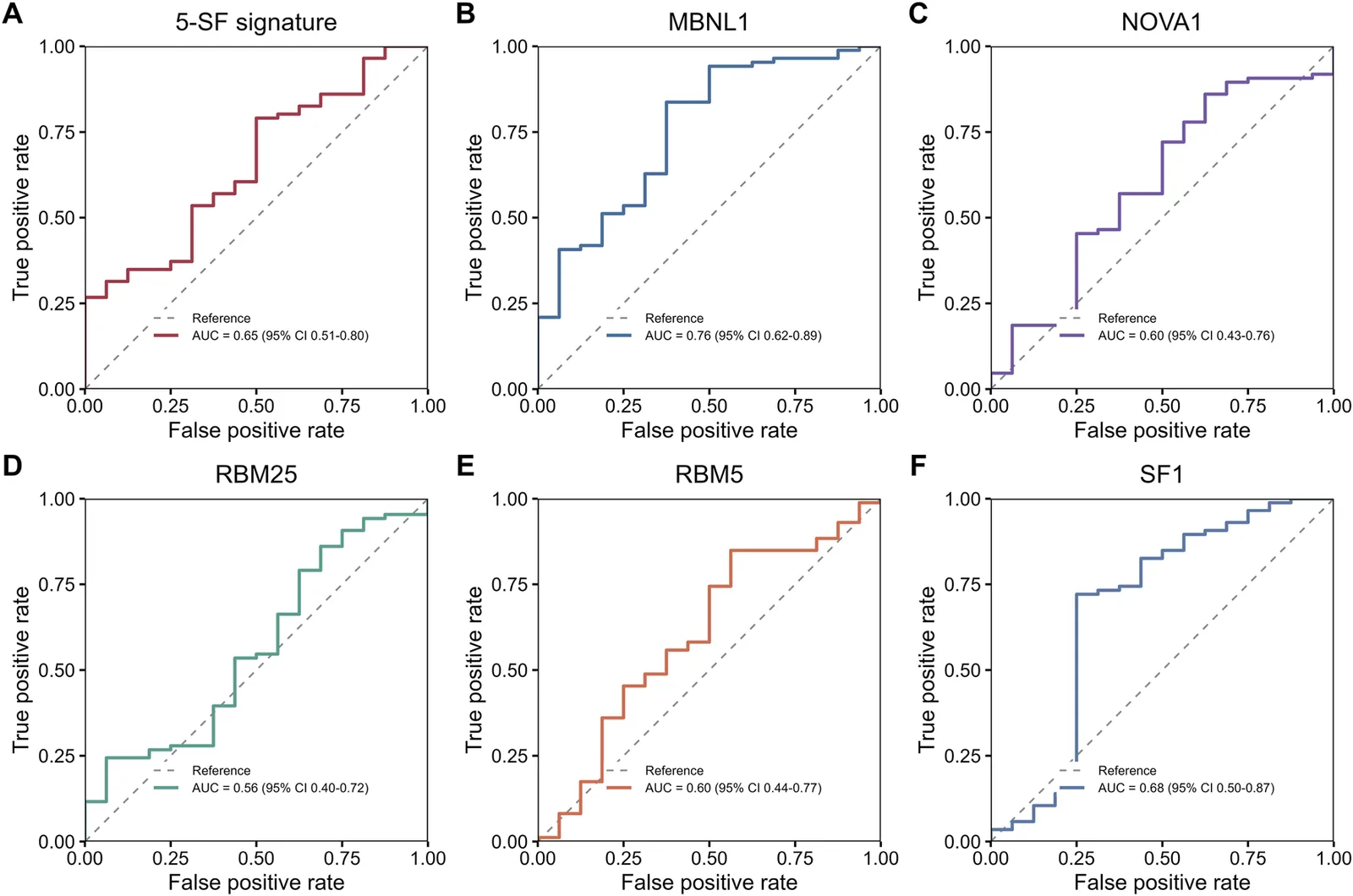
External dataset (GSE5406) validation of characteristic splicing factors in DCM. **(A)** ROC curve evaluating the diagnostic performance of the combined five-SF signature. **(B–F)** ROC curves showing the diagnostic performance of individual characteristic splicing factors, including *MBNL1* **(B)**, *NOVA1* **(C)**, *RBM25* **(D)**, *RBM5* **(E)**, and *SF1* **(F)**. AUC values and 95% confidence intervals are shown in each panel.

Model evaluation constitutes an essential component in building effective machine learning models. In this study, we employed receiver operating characteristic (ROC) analysis to validate the predictive accuracy of our model using an independent external dataset. The area under the ROC curve (AUC) provides a quantitative measure of classification performance, where higher values indicate superior discriminative ability. Our investigation identified distinctive splicing factors associated with DCM pathogenesis, findings that were consistently replicated across multiple validation cohorts. Particularly in the GSE5406 dataset, ROC analysis demonstrated the diagnostic potential of these characteristic splicing factors in DCM stratification. The AUC values with 95% confidence intervals for *NOVA1*, *MBNL1*, *RBM5*, *RBM25*, and *SF1* were 0.60 (95% CI: 0.43–0.76), 0.76 (95% CI: 0.62–0.89), 0.60 (95% CI: 0.44–0.77), 0.56 (95% CI: 0.40–0.72), and 0.68 (95% CI: 0.50–0.87), respectively. The five-SF signature achieved an AUC of 0.65 (95% CI: 0.51–0.80), and these diagnostic performance metrics are illustrated in Figures 12A–F. GSE3586 was analyzed separately as a same-study cross-platform replication cohort and is shown in Supplementary Figure S2.

## Discussion

This study elucidates the critical role of splicing factor-mediated regulatory networks and immune dysregulation in the pathogenesis of DCM. By integrating RNA-seq data with three machine learning methods including LASSO regression, Random Forest, and SVM-RFE, we identified five SFs including *MBNL1*, *NOVA1*, *RBM25*, *RBM5*, and *SF1* as characteristic splicing factors related to immune microenvironment alterations in DCM. Notably, several of these SFs, such as *MBNL1* and *RBM25*, have previously been implicated in cardiac development and dysfunction, supporting their potential mechanistic relevance. The consensus clustering analysis stratified DCM patients into two distinct clusters with divergent immune signatures, characterized by differences in cytolytic activity, Type I interferon response, and antigen-presenting cell (APC) costimulatory pathways, suggesting that these subtypes might highlights the heterogeneity of DCM and suggests that dysregulated splicing may drive unique immunological endotypes, which could inform personalized therapeutic strategies. Furthermore, utilizing weighted gene co-expression network analysis and PPI network, hub genes such as *LAMP2* and *COPZ1*, associated with autophagy and ribosomal function, and *KIAA0196*, *MRPS14*, *RPS15A*, and *GNPAT* were identified as potential mediators of cardiac dysfunction. These findings extend current knowledge by linking SF dysregulation to immune remodeling, a mechanism previously underexplored in DCM (Shi, 2024; Zhang et al., 2022). These findings underscore the pivotal role of splicing factor-mediated regulatory networks and immune dysregulation in the pathogenesis of DCM, advance the mechanistic understanding of DCM pathogenesis by establishing the correlation between splicing factors dysregulation and immune microenvironment. Nevertheless, given the relatively small and imbalanced cluster sizes, the stability and generalizability of the MEblack–cluster A association require further validation in larger independent cohorts.

The incorporation of RNA-seq-based splicing analysis substantially broadens the scope of this study from splicing factor expression profiling to transcript isoform remodeling at the event level. Rather than reflecting a uniform shift in exon usage, DCM myocardium exhibited widespread and bidirectional PSI alterations across multiple classes of alternative splicing events. The clear separation of DCM and control samples based on DAS-derived PCA and heatmap patterns further supports the existence of a coordinated, disease-associated splicing program. Importantly, functional enrichment and representative junction-count schematics linked these altered events to key myocardial processes, including cardiac contraction, calcium handling, sarcomeric and cytoskeletal organization, and RNA processing. Collectively, these findings indicate that the characteristic splicing factor signature is embedded within a broader and functionally relevant alternative splicing landscape in DCM, rather than representing an isolated expression pattern.

The characteristic SF-AS network further provides a structured framework for linking candidate splicing regulators with disease-relevant splicing events. By integrating splicing factor expression with DAS-derived PSI changes, this network highlights coordinated relationships between regulatory factors and transcript-level remodeling. As such, it offers a systems-level view that connects splicing factor dysregulation to functional transcriptomic alterations in DCM, thereby reinforcing the concept that aberrant splicing regulation constitutes an integral component of disease-associated molecular remodeling.

The coatomer protein complex subunit zeta 1 (COPZ1), a key component of the coat protein complex I, plays a significant role in various cellular processes, including intracellular transport, endosome development, lipid balance, and autophagy. Notably, COPZ1 influences iron metabolism by regulating transferrin. Additionally, it contributes to maintaining hepcidin levels, which are crucial for controlling iron entry into the bloodstream of mammals (Beck et al., 2009; Razi et al., 2009; Collinet et al., 2010; Mleczko-Sanecka et al., 2014).

The *KIAA0196* gene, encodes a pan-tissue expressed scaffolding protein that demonstrates elevated expression in musculoskeletal and neural systems. As the structural scaffold of the WASH (Wiskott-Aldrich Syndrome Protein and SCAR Homolog) regulatory complex, this multidomain protein orchestrates actin cytoskeleton remodeling through Arp2/3-mediated nucleation, critical for endosomal sorting processes (Seaman, 2005; Cullen and Korswagen, 2011). Emerging evidence links loss-of-function variants in the WASHC5 locus (encoding strumpellin) to cardiovascular pathogenesis. Clinical cohorts revealed that heterozygous carriers exhibit a 60% reduction in strumpellin expression accompanied by structural cardiac anomalies (Elliott et al., 2013). Experimental models recapitulate this phenotype: haploinsufficiency of this genetic locus in zebrafish and murine systems induces pericardial effusion or cardiomegaly, while complete ablation induces embryonic inviability underscoring its evolutionarily conserved cardioprotective role through disrupted actin polymerization dynamics (Valdmanis et al., 2007; Clemen et al., 2010).

RPS15A (Ribosomal Protein S15A), a conserved component of the 40S ribosomal subunit encoded by the 16p12.3 genomic locus, plays dual roles in canonical and non-canonical biological processes. Mechanistic studies reveal its critical involvement in translation initiation by facilitating cap-dependent mRNA recruitment to the small ribosomal subunit. Beyond its structural role in ribosome biogenesis, this multifunctional protein directly interacts with calcium/calmodulin (Ca^2+^/CaM) complexes to regulate translation-coupled calcium signaling (Shen et al., 2005). Beyond its canonical role in ribosome biogenesis, accumulating evidence suggests that RPS15A shares various extra-ribosomal functions with other members of the RPS family, including roles in cell division, tumorigenesis (Jiménez et al., 2004).

Our results align with prior studies emphasizing the role of RNA-binding proteins (RBPs) like RBM20 and RBM24 in DCM pathogenesis through sarcomeric gene splicing, particularly in TITIN isoform switching (Shi, 2024). For example, RBM20 mutations disrupt cardiac stiffness by altering TITIN splicing, leading to ventricular dilatation—a hallmark of DCM (Shi, 2024). Similarly, our identification of RBM25 and RBM5 as critical SFs suggests broader roles for RBPs beyond TITIN regulation, potentially impacting calcium handling or mitochondrial function. Notably, the immune dysregulation observed in our SF-based clusters parallels reports of autophagy-related gene networks in DCM, where LAMP2 (a lysosomal protein) and VDAC1 (a mitochondrial channel) influence cardiomyocyte survival and stress responses (Deacon et al., 2019). LAMP2, a gene encoding lysosomal membrane-associated proteins, plays critical roles in cellular processes. Pathological conditions linked to this genetic locus encompass Danon cardiomyopathy (Endo et al., 2015) and ventricular trabeculation abnormalities (Left Ventricular Noncompaction). Functionally significant biological pathways involving LAMP2 include calcium-mediated platelet activation mechanisms and fundamental pathogen defense networks. Molecular interactions characterized for this gene product demonstrate specific affinity for catalytic biomolecules and selective recognition of particular protein domains (Tanaka et al., 2000). This synergy between splicing errors and immune activation may exacerbate ventricular remodeling, a hypothesis supported by studies linking inflammatory pathways to DCM progression. The relationship between AS remodeling and immune activation in DCM may be bidirectional. DAS events involving *TTN*, *RYR2*, *LDB3*, and *FLNA* may disrupt myocardial contraction, calcium handling, and cytoskeletal integrity, thereby inducing cardiomyocyte stress and secondary inflammation. Conversely, inflammatory signaling may alter splicing-factor activity and isoform usage. Therefore, these processes may interact with each other or occur in parallel during DCM progression, although their causal relationship requires further functional validation.

Several limitations should be acknowledged. The candidate biomarkers were derived from myocardial tissue and should therefore be considered tissue-based molecular biomarkers requiring further validation in prospective cohorts and accessible biospecimens, particularly peripheral blood. Incomplete clinical information prevented adjustment for relevant covariates. Additionally, the small sample size, particularly Cluster B (n = 7), warrants cautious interpretation of the clustering and between-cluster comparisons and requires validation in larger independent cohorts. The bulk nature of the analyzed myocardial datasets precludes direct assignment of gene expression or splicing events to specific cell types. Differences in inferred immune enrichment, SF expression, and PSI may therefore partly reflect changes in cardiomyocyte, stromal, endothelial, or immune-cell proportions. Cell-type-resolved and spatial transcriptomic studies will be required to localize the observed alterations.

## Conclusion

In conclusion, this study provides an integrated landscape of splicing factor dysregulation, alternative splicing remodeling, immune perturbation, and molecular heterogeneity in dilated cardiomyopathy (DCM). By coupling machine learning-based feature selection with external validation, we identified five candidate feature SFs—*MBNL1*, *NOVA1*, *RBM25*, *RBM5*, and *SF1*—with *MBNL1* receiving the strongest external diagnostic support. Beyond expression profiling, event-level analysis identified widespread differential alternative splicing events in the DCM myocardium, involving genes associated with cardiac function and related biological pathways. The construction of an SF–AS regulatory network linked these splicing factors to percent spliced in (PSI) alterations in disease-associated AS events, providing an association-based framework for understanding how splicing factor dysregulation shapes transcript isoform remodeling in DCM. Concurrently, molecular subtype analysis identified distinct DCM subgroups characterized by divergent immune infiltration patterns, pathway activities, and transcriptomic signatures. Key hub genes, including *COPZ1*, *LAMP2*, *KIAA0196*, *MRPS14*, *RPS15A*, and *GNPAT*, may represent downstream molecular mediators and candidate therapeutic targets associated with subtype-specific remodeling. Collectively, these findings bridge AS dysregulation, characteristic splicing factor signatures, immune remodeling, and molecular heterogeneity within a unified model of DCM pathobiology, offering promising candidate tissue-level biomarkers and mechanistic candidates for future experimental validation.

## Data Availability

The datasets analyzed during the current study are available in the Gene Expression Omnibus (GEO) repository. The raw expression data of dilated cardiomyopathy analyzed in this study were downloaded with the following accession numbers GSE116250: (https://www.ncbi.nlm.nih.gov/geo/query/acc.cgi?acc=GSE116250) GSE3585 (https://www.ncbi.nlm.nih.gov/geo/query/acc.cgi?acc=GSE3585), GSE3586 (https://www.ncbi.nlm.nih.gov/geo/query/acc.cgi?acc=GSE3586), GSE42955 (https://www.ncbi.nlm.nih.gov/geo/query/acc.cgi?acc=GSE42955), GSE79962 (https://www.ncbi.nlm.nih.gov/geo/query/acc.cgi?acc=GSE79962).
