# Supplementary material for: Alternative splicing remodeling and splicing factor regulatory networks define immune-associated molecular heterogeneity in dilated cardiomyopathy
Source: Front Bioinform. 2026 Sep 4;6:1934275. doi: 10.3389/fbinf.2026.1934275 (PMC13585734; doi:10.3389/fbinf.2026.1934275)
Supplement: Supplementary file 1 [file DataSheet1.zip › Supplementary Information.pdf]

Supplementary Information

Supplementary Data 1. Expression profiles of 24 splicing factors in dilated cardiomyopathy and control myocardium.

Normalized transcript abundance of 24 splicing factors across study cohorts used for differential expression analysis and integrative modeling.

Supplementary Data 2. Gene module annotation derived from weighted gene co-expression network analysis (WGCNA).

Module assignments of genes identified through co-expression network construction for downstream systems-level analysis.

Supplementary Data 3. Immune cell enrichment scores estimated by single-sample gene set enrichment analysis (ssGSEA).

Quantitative immune cell infiltration profiles used to characterize immune microenvironment heterogeneity.

Supplementary Data 4. Immune functional activity scores estimated by single-sample gene set enrichment analysis (ssGSEA).

Enrichment scores representing immune-related functional states applied for comparative immunological assessment.
